# Downsizing in total hip arthroplasty. A short stem as a revision implant

**DOI:** 10.1007/s00132-021-04168-8

**Published:** 2021-09-28

**Authors:** Marcel Coutandin, Yama Afghanyar, Philipp Rehbein, Jens Dargel, Philipp Drees, Karl Philipp Kutzner

**Affiliations:** 1 Department of Orthopaedics and Traumatology, St. Josefs Hospital Wiesbaden, Beethovenstr. 20, 65189 Wiesbaden, Germany; 2 Department of Orthopaedics and Traumatology, University Medical Centre of the Johannes Gutenberg-University of Mainz, Langenbeckstraße 1, 55131 Mainz, Germany

**Keywords:** Revision surgery, Hip replacement, Short stem, De-escalation, Optimys, Revisionsoperation, Hüftgelenkersatz, Kurzschaft, Deeskalation, Optimys

## Abstract

**Background:**

Short stems have constantly gained popularity in primary total hip arthroplasty (THA) over the last decade. Although cementless short stems are not primarily designed to be used as revision implants, there may be certain indications for which downsizing the femoral component in failed conventional THA is potentially advantageous.

**Methods:**

In this single center retrospective case series, six patients who underwent revision using a calcar-guided short stem after failed THA are presented. The mean follow-up was 3.32 years (SD 0.63 years). The health status was evaluated by the EQ-5D-5L score. Patient reported outcome measurements (PROM) were recorded using the Harris hip score (HHS) and The Western Ontario and McMaster Universities Osteoarthritis Index (WOMAC). Pain and satisfaction were assessed using a visual analogue scale (VAS). Radiographic analysis was performed by evaluating osteolysis, stress shielding, alignment and signs of aseptic loosening. Complications were documented.

**Results:**

At last follow-up the mean EQ-5D-5L index was 0.851 (SD 0.098). Clinical outcome was excellent (HHS ≥ 90) in 4 patients and moderate (HHS 71 and 79) in 2 patients. The mean WOMAC score was 9.20% (SD 12.61%). Pain and satisfaction on VAS were 1.00 (SD 1.15) and 9.17 (SD 0.37), respectively. No major complications occurred. To date, no further revision surgery was needed. Radiologically, no signs of subsidence, aseptic loosening, stress shielding and fractures were obvious.

**Conclusion:**

The present case series indicates that in failed conventional THA downsizing may be considered a treatment option, using short stem THA in selected cases.

## Abstract (de)

**Hintergrund:**

Kurzschäfte haben in der primären Hüfttotalendoprothetik (Hüft-TEP) im letzten Jahrzehnt stetig an Popularität gewonnen. Obwohl zementfreie Kurzschäfte nicht primär für den Einsatz als Revisionsimplantate konzipiert sind, stellt das „Downsizing“ der Femurkomponente bei fehlgeschlagener konventioneller Hüft-TEP bei ausgewählten Indikationen potenziell eine Behandlungsalternative dar.

**Methoden:**

In dieser retrospektiven Fallserie werden 6 Patienten vorgestellt, die nach einer fehlgeschlagenen Hüft-TEP unter Nutzung eines kalkargeführten Kurzschafts revidiert wurden. Die mittlere Nachbeobachtungszeit betrug 3,32 Jahre (SD 0,63). Der Gesundheitszustand wurde mit dem EQ-5D-5L-Score bewertet. Die von den Patienten berichteten Ergebnisse (PROMs) wurden mit dem Harris Hip Score (HHS) und dem Western Ontario and McMaster Universities Osteoarthritis Index (WOMAC) erfasst. Schmerz und Zufriedenheit wurden mit Hilfe der visuellen Analogskala (VAS) erfasst. Die radiologische Analyse erfolgte durch die Bewertung hinsichtlich, Sinterung, Osteolysen, „stress shielding“ und Anzeichen von aseptischer Lockerung. Komplikationen wurden dokumentiert.

**Ergebnisse:**

Beim letzten Follow-up betrug der mittlere EQ-5D-5L-Index 0,851 (SD 0,098). Die klinischen Ergebnisse waren bei 4 Patienten ausgezeichnet (HHS ≥ 90) und bei 2 Patienten befriedigend (HHS 71 und 79). Der mittlere WOMAC-Score betrug 9,20 % (SD 12,61). Schmerz und Zufriedenheit auf der VAS lagen bei 1,00 (SD 1,15) bzw. 9,17 (SD 0,37). Es traten keine größeren Komplikationen auf. Bis heute war keine weitere Revisionsoperation erforderlich. Radiologisch zeigten sich keine Anzeichen von Sinterung, aseptischen Lockerungen, „stress shielding“ und Frakturen.

**Schlussfolgerung:**

Die vorliegende Fallserie legt nahe, dass bei fehlgeschlagener konventioneller Hüft-TEP das „Downsizing“ als Behandlungsoption in ausgewählten Fällen in Betracht gezogen werden kann.

## Introduction

Cementless and cemented conventional femoral stems have been proven to be successful in total hip arthroplasty (THA) for patients with osteoarthritis of the hip [22]. Data for long-term survival (> 95%) of conventional stems at 10 years postoperatively can be found in both national registries as well as case series [12, 13]; however, THA revision rates have grown steadily in recent years, due to increased life expectancy in a globally aging population [18]. Common causes of revision THA are aseptic loosening due to wear and infection [14].

Frequently required revision procedures lead to technically highly demanding surgery, often associated with complications. In those situations, particularly in older patients with severe comorbidities, often the desired surgical result has to be weighed against the surgical trauma, the damage to the bone stock and the patient’s well-being by the surgeon. This depends on multiple factors, including surgeon’s level of experience, previous approach, reason for revision, patient’s characteristics and the type of implant requiring removal.

Potential reasons for highly challenging femoral revision procedures are insufficient bone stock, remaining metal parts of the primary implants as well as remaining cement and sclerotic bone formation in the medullary canal.

Surgical options often constitute a therapeutic escalation, i.e. a complexification of treatment sometimes related to the unnecessary use of long revision stems as well as cement [27].

Cementless short stems have gained in popularity in recent years. They were initially designed to achieve a more anatomical pattern of stress distribution by loading the femur proximally [21]. Additionally, short stems claim several further potential advantages, including soft tissue preservation, enhanced proximal bone remodeling, less blood loss, shortened postoperative rehabilitation and recovery and simplified femoral revisions [19, 21, 26].

Applying a short stem as a revision implant could potentially reduce the perioperative secondary surgical risks, for example by avoiding femoral osteotomy and a transfemoral approach, which is often necessary for the removal of parts of the implant, remaining cement and sclerotic bone formation from the medullary canal [7].

Thus, there may be certain assorted indications, for which downsizing the femoral component in failed conventional THA is potentially advantageous. To our best knowledge, only one case report of revision surgery of conventional hip arthroplasty using a cementless short stem has been published so far [7].

Therefore, the aim of this case series was to introduce the concept of downsizing and to investigate short-term clinical results and complications of revision surgery of failed conventional THA using a short stem in assorted indications.

## Material and methods

**Fig. 1:**
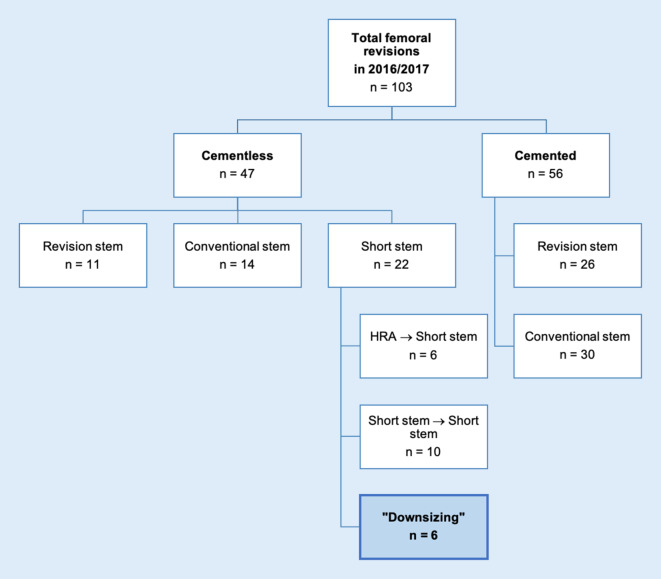
Flow diagram of the patients included in the study. *HRA* Hip resurfacing arthroplasty

This research has been approved by the IRB of the authors’ affiliated institutions. Written informed consent has been obtained by all patients. This is a retrospective case series with six patients included, for whom revision surgery of failed conventional hip arthroplasty was performed using short-stem THA in the years 2016 and 2017 at a single institution (Table 1). During that time, a total of 103 patients underwent revision THA including femoral revision. Figure 1 shows the flow diagram of the patients included in the study.

**Table 1:** **Patient characteristics**

	Year (index surgery)	Failed stem	Side	Gender	Age (years, at revision)	BMI	Paprosky	Indication
Pat. 1	2006	Marathon (Smith&Nephew, Watford, UK)	Left	Male	82	30.5	II	Aseptic loosening
Pat. 2	2013	Revitan (Zimmer Biomet, Warsaw, IN, USA)	Left	Male	65	38.6	II	Implant fracture
Pat. 3	2015	CLS Spotorno (Zimmer Biomet, Warsaw, IN, USA)	Right	Male	63	25.1	I	Aseptic loosening
Pat. 4	2016	MEM (Zimmer Biomet, Warsaw, IN, USA)	Left	Male	82	22.1	IIIa	Periprosthetic infection
Pat. 5	2011	Rippenschaft (Link, Hamburg, Germany)	Left	Male	77	25.7	I	Aseptic loosening
Pat. 6	2000	ABG 2 (Stryker, Kalamazoo, MI, USA)	Left	Male	72	23.3	II	Aseptic loosening

*BMI* body mass index

The index procedures of the investigated collective were performed between 2000 and 2016 in different hospitals. Mean time before revision was 6.68 ± 5.82 years (range 1.25–17.75 years). The indications for revision surgery were aseptic loosening (66.67%, *n* = 4), fracture of the implant (16.67%, *n* = 1) and periprosthetic infections (16.67%, *n* = 1), providing loss of function and severe pain (*n* = 6). They were all males (*n* = 6) and mean patient age was 73.5 years (range 63–82 years).

Preoperative anteroposterior imaging was performed and the amount of bone loss was scored according to the Paprosky classification [34].

All procedures were performed using an anterolateral approach. Bone loss was reassessed after component removal, again according to Paprosky.

**Fig. 2:**
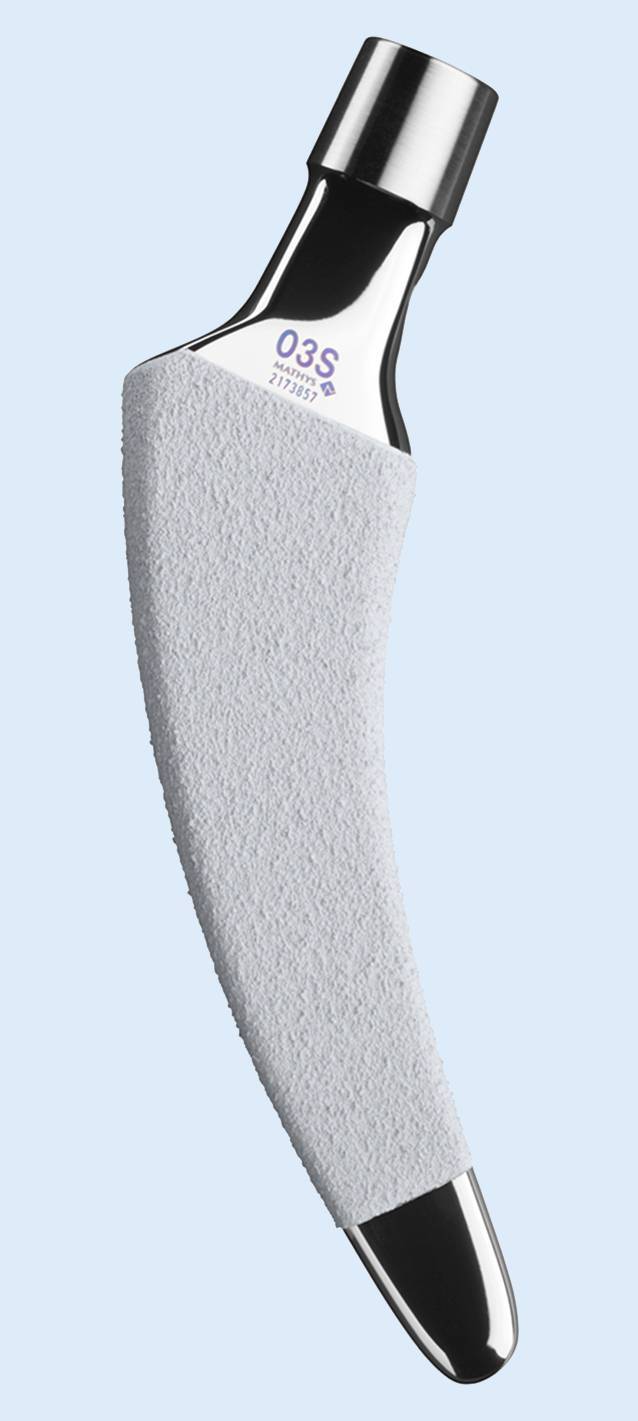
The optimys short stem (With kind permission © MathysAG Bettlach, Switzerland, all rights reserved)

In all patients the calcar-guided short stem optimys (Mathys. Bettlach, Switzerland) was used as a revision implant (Fig. 2).

For the acetabular component either a cementless primary press-fit cup or a revision cup was used. Whereas in few cases full weight bearing was permitted, most patients required partial weight bearing. Postoperatively, again anteroposterior imaging was performed during follow-up.

For health status, the EQ-5D-5L (EuroQol Group) was used [5]. Patient reported outcome measurements (PROMs) were obtained at last follow-up, such as the Harris hip score (HHS; range from ≥ 90 = excellent to < 70 = poor), the Western Ontario and McMaster Universities Osteoarthritis Index (WOMAC; range from 0% = best to 100% = worst) as well as pain (0 = no pain to 10 = worst pain possible) and satisfaction (0 = worst to 10 = best) on a visual analogue scale (VAS).

All statistical analyses were performed using Excel (Microsoft). Data are reported by mean, standard deviation (SD) and range.

## Results

Between 2016 und 2017, six patients underwent revision THA using a calcar-guided short stem optimys (Mathys). The mean clinical and radiological follow-up was 3.32 ± 0.63 years (range 2.6–4.2 years) and the mean age at revision surgery was 73.5 ± 7.54 (range 63–82 years). The main selection criteria for downsizing to a short stem were the presence of remaining cement and sclerotic bone formation in the medullary canal, and at the same time sufficient proximal bone stock in order to obtain good primary stability. In some of the cases, the decision was made only during the procedure, despite deviating from the preoperative planning.

Before extraction of the loosened femoral components (in one case the femoral spacer), the amount of bone loss was graded as Paprosky type I for 2 cases, type II for 3 cases and type IIIa for 1 case.

A short description of each case is shown below.

### Patient 1 (Fig. 3a–c).

An 82-year-old male presented with increasing pain in the left groin and progressive leg shortening. Initially cemented THA was performed in 2006. The patient was diagnosed with aseptic loosening of the acetabular and femoral components. A cementless revision cup was combined with an acetabular bone plastic. On the femoral side, after removal of the loosened stem, distal parts of the cement remained in the medullary canal and were not to be removed easily without osteotomy and a transfemoral approach. Given sufficient proximal bone stock, the decision was taken to leave the distal parts of the remaining cement in place and to implant the cementless short stem.

**Fig. 3:**
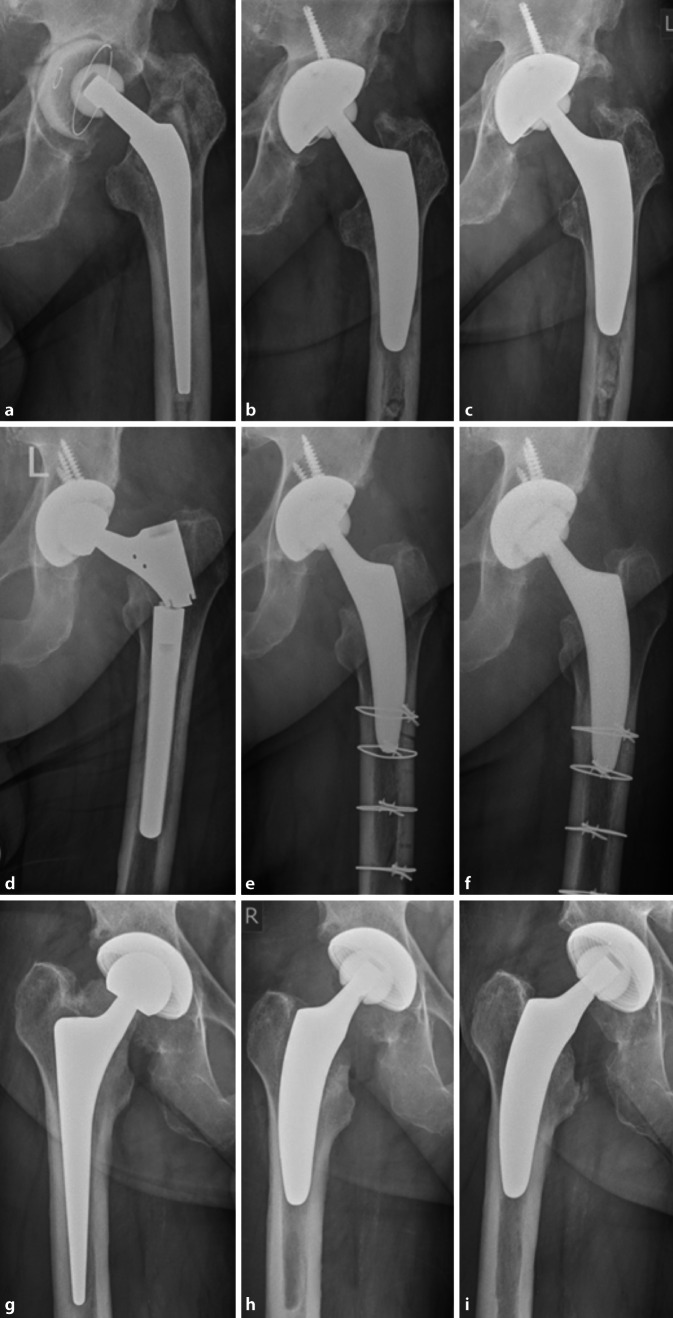
Radiographs of the hip joint of patients 1 (**a**–**c**), 2 (**d**–**f**) and 3 (**g**–**i**), **a,d,g** preoperatively, **b,e,h** postoperatively, **c,f,i** at last follow-up

### Patient 2 (Fig. 3d–f).

A 65-year-old male who presented at the emergency room with sudden acute pain and immediate immobility. The index procedure was performed in 2012. Due to periprosthetic infection, a two-stage revision was performed in 2013 using a modular, cementless stem. The imaging revealed implant breakage. Revision surgery involved a transfemoral approach to remove the well-integrated distal part of the stem and subsequent cerclage wiring. As the cementless revision stem was not found to provide rotational stability intraoperatively, a trial was done using the cementless optimys short stem. Since the rather young patient provided sufficient metaphyseal bone stock, a good press-fit was achieved. Additionally, the polyethylene inlay was revised. The patient was treated with partial weight bearing using crutches for 6 weeks.

### Patient 3 (Fig. 3g–i).

A 63-year-old male whose symptoms had deteriorated with strong pain on the right hip along with progressive leg shortening. Initially conventional cementless THA was performed in 2015. As no evidence for an infection was found, the patient was diagnosed with aseptic loosening and marked subsidence. At the same time, the patient suffered from symptoms of osteoarthritis on the left hip. Given the young age of the patient and almost perfect bone quality in the proximal femur, revision was successfully performed using the optimys short stem. At the same stage, primary short stem THA was performed on the opposite side.

### Patient 4 (Fig. 4a–c).

An 82-year-old male presented with periprosthetic infection following cemented THA in 2016. Revision surgery involved a two-stage strategy with explanation and spacer implantation, combined with antimicrobial therapy. Femoral reimplantation was planned using a cementless revision stem; however, intraoperatively, due to remaining cement distally, the broach went *via falsa* several times. Due to the high age and several comorbidities, the decision was taken not to use a transfemoral approach, but to perform a trial with the cementless short stem. Finally, an optimys stem combined with a cemented revision cup was implanted. Following revision surgery, the patient was allowed only partial weight bearing.

**Fig. 4:**
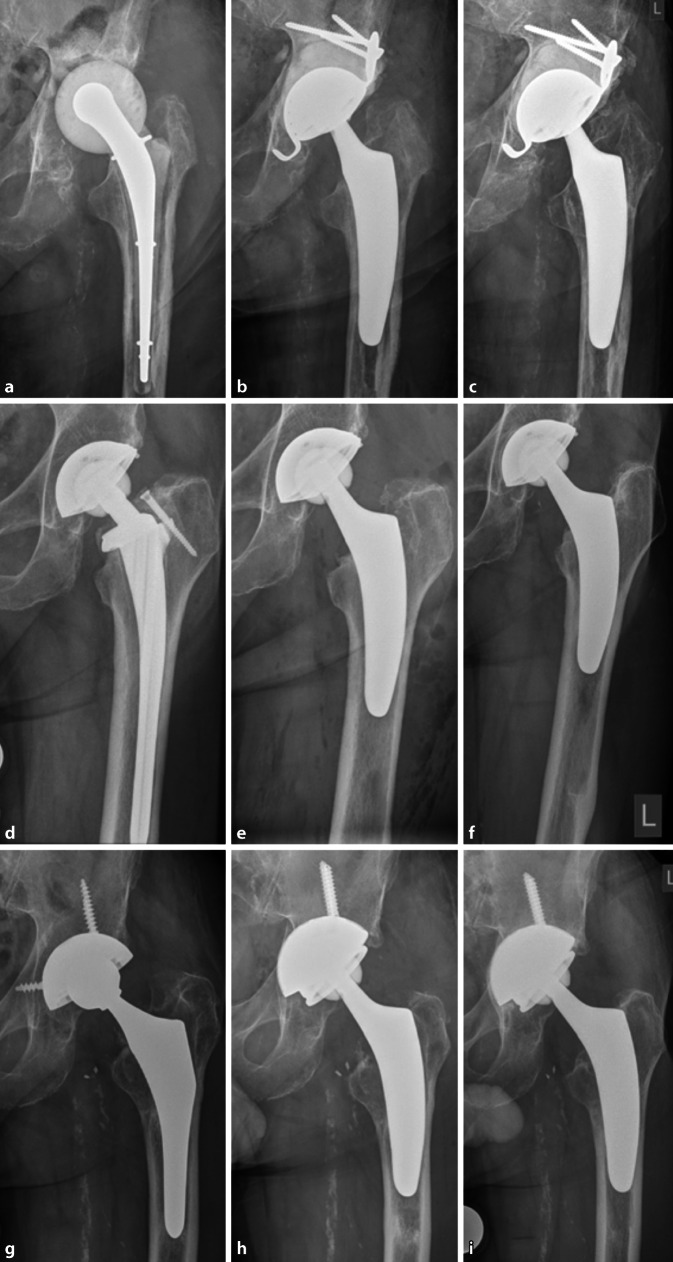
Radiographs of patients 4 (**a–c**), 5 (**d–f**) and 6 (**g**–**i**), **a,d,g** preoperatively, **b,e,h** postoperatively, **c,f,i** at last follow-up

### Patient 5 (Fig. 4d–f).

A 77-year-old male presented with increasing pain and progressive leg shortening. Initially THA was performed in 2011 using a cementless conventional stem with collar. After having ruled out periprosthetic infection, aseptic loosening with subsidence of the femoral component was diagnosed. Given sufficient bone quality in the metaphyseal proximal femur, revision was performed using the optimys short stem. The patient was allowed full weight bearing.

### Patient 6 (Fig. 4g–i).

A 72-year-old male whose symptoms had slowly deteriorated with pain following cementless conventional THA in the year 2000. The patient was diagnosed with aseptic loosening. The acetabular component was revised using a cementless revision cup, because inlays had not been available for the primary cup. During femoral preparation, even reamers would not penetrate the sclerotic formation found in the diaphysis. Given the marked danger of cortical damage, the decision was taken to perform a trial using the optimys short stem. As the bone quality in the proximal femur was sufficient and sclerotic, a good press-fit could be achieved. The patient was instructed to partial weight bearing for 4 weeks.

The clinical outcomes are summarized in Table 2.

**Table 2:** Clinical outcomes

	Follow-up (years)	HHS	WOMAC (In %)	EQ-5D-5L (Index)	Pain (VAS)	Satisfaction (VAS)
Pat. 1	4.0	71	21.9	0.738	1	9
Pat. 2	4.2	96	1.0	0.910	2	9
Pat. 3	3.5	100	0.0	0.909	0	9
Pat. 4	3.0	79	31.3	0.723	0	9
Pat. 5	2.6	96	1.0	0.828	3	9
Pat. 6	2.6	100	0.0	1.000	0	10

*HHS* Harris Hip Score, *WOMAC* Western Ontario and McMaster Universities Osteoarthritis Index, *EQ-5D-5L* health status by the EuroQol Group, *VAS* visual analogue scales

Mean HHS was 90.33 ± 11.21 (range 71–100), the outcome of 4 patients was excellent (HHS ≥ 90), except for 2 patients, which was moderate (HHS 71 and 79). The mean WOMAC score was 9.20% ± 12.61% (range 0.0–31.3%). Mean pain on VAS was 1.00 ± 1.15 (range 0–3) and mean satisfaction on VAS was 9.17 ± 0.37 (range 9–10).

No further revision surgery has been necessary so far. During follow-up, no major complications occurred. No patient died before the final follow-up. Radiologically, no signs of subsidence, aseptic loosening, stress shielding and fracture were obvious (Figs. 3 and 4).

## Discussion

With the number of primary THA continuously growing, surgeons will inevitably be confronted with the challenge of a rising number of revision procedures as well [18, 25]. As patients become increasingly younger of age at the time of primary THA, they most likely will experience more than one revision surgery during their lifetime [29]. In contrast to primary THA, revision surgery is technically more demanding, often requiring extensive surgical exposure and careful management of periprosthetic bone loss. Although the usage of short stems in revision THA is to be considered off-label use, in order to reduce the surgical trauma as well as to save as much femoral bone stock as possible, in assorted cases, “downsizing” the femoral component may be considered. This present case series aimed to introduce the concept of “downsizing” and to investigate the outcome of revision THA using a short stem in patients with failed conventional THA.

Success in achieving and maintaining stable implant fixation following revision THA is dependent upon component design, surgical technique and pre-existing damage to the bone stock [6]. Over the last decade, there was no consensus on whether cementless or cemented revision stems are the best choice in femoral revision surgery. Ultimately, the choice of fixation method in revision surgery is still a matter not only of science and evidence, but also of preference and local tradition [33].

However, since cemented stems in revision THA were reported to have unacceptably high rates of mechanical failure at early and mid-term follow-up during the 1980s [1, 4], there was a trend towards cementless femoral revision implants in several countries [11, 13].

Regarding femoral revision, in general, the objective of replacing the initial stem by another with a “fixation as proximal as possible and as distal as necessary” should be pursued [2]. Cementless modular revision stems offer the option of distal anchoring within intact bone; however, in some cases, diaphyseal conditions do not allow unproblematic distal anchoring. Potential reasons include conflicting intramedullary implants, remaining cement, deformity and diaphyseal bone defects.

A more proximal canal fill and cementless biologic ingrowth may provide sufficient implant stability but avoid proximal stress shielding of the femur and improve long-term implant survival in the revision situation [10]. Using a proximally anchoring stem bears the potential of subsequent proximal bone remodeling.

Another main advantage of the approach of using a proximally anchoring cementless stem in assorted situations, compared with diaphyseal-fitting stems, is the simplicity of the procedure because diaphyseal reaming is not required, leading to a preservation of the bone stock. Diaphyseal reaming may account for the high perioperative femoral fracture rates reported by different authors. For example, Nadaud et al. reported an incidence of 13% [24]. In the present case series at least in one case there was high danger of intraoperative fracture in the process of the removal of the remaining cement. Partial damage caused by *via falsa* preparation led to the decision of choosing the proximally anchoring short stem as a salvage procedure.

In 2012, Miletic et al. reported on the concept of de-escalation, which involved changing a long revision stem to a standard length cementless or cemented conventional stem [23]. At a mean follow-up of 55 months, no signs of failure were seen and none of the patients required additional surgery. The authors concluded that de-escalation exchange of a failed locked revision stem with a shorter stem is a feasible option.

To date, several studies have investigated the outcomes of femoral revision using a primary conventional cementless stem. An overview is provided in Table 3 [3].

**Table 3:** Overview of studies investigating primary conventional cementless stems used as a revision implant

Study	Implant	N (hips)	Follow-up (years)	Survival (%)
Tauber et Kidron, 2000 [30]	CLS Spotorno (Zimmer Biomet, Warsaw, IN, USA)	24	4.5	96
Kelly et al., 2006 [15]	Securfit plusTM (Stryker, Kalamazoo, MI, USA)	32	5	91
Thorey et al., 2008 [32]	Bicontact (BBraun Aesculap, Melsungen, Germany)	79	7	95
Salemyr et al. 2008 [28]	Bi-Metric (Zimmer Biomet, Warsaw, IN, USA)	62	6.1	93.6
Pinaroli et al., 2009 [27]	Corail (Depuy Synthes, Raynham, MA, USA)	41	2.5	100
Miletic et al., 2012 [23]	Alloclassic (Zimmer Biomet, Warsaw, IN, USA)	15	4.5	100
Tetreault et al., 2014 [31]	Various	144	4	90.2
Khanuja et al., 2014 [17]	Accolade TMZF (Stryker, Kalamazoo, MI, USA)	19	5	94.8
Gastaud et al., 2016 [9]	Linea (Tornier, Burscheid, Germany)	43	4	100

However, to date, almost no data are available regarding revision of conventional THA using a short stem. Evola et al. recently published a case report of one patient with stem breakage in the distal part. Intraoperatively, the distal apex of the implant could not be removed easily [7]. In this situation, to avoid a transfemoral approach, extensive operative time associated with increased blood loss and marked soft-tissue damage, a Fitmore short stem (Zimmer; Winterthur, Switzerland) was used. To obtain primary stability, the authors chose a cemented fixation of the cementless component. The 2‑year follow-up resulted in a good clinical outcome with stable implant position. They concluded that short-stem designs can help surgeons to treat specific revision procedures in patients with poor general health conditions to avoid a surgical invasiveness due to transfemoral approaches and long-stemmed revision implants [7].

Gamboa et al. [8] reported an uncommon scenario in which options for femoral fixation in primary THA were limited as the femoral diaphysis was almost completely filled by a long-stemmed revision knee replacement. Preoperative templating showed that conventional THA could not be accommodated and therefore a short stem was selected. As the patient was neither young nor active and had osteoporosis, the decision was controversial; however, confronted with limited high-risk options, also this case demonstrates the successful use of a short stem in the presence of inadequate femoral bone stock as a consequence of previous surgery or deformity [8].

In the present investigation, a variation of indications led to the usage of a cementless short stem as a revision implant. Most cases presented with aseptic loosening and subsequent migration of the primary femoral implants. In one case a periprosthetic infection and in another case femoral implant breakage were diagnosed. In most cases, the bone quality in the proximal femur was good. The formation of sclerotic bone in the metaphysis, due to micromotion of the primary implants, often allows for sufficient cementless press-fit anchoring.

The results of the present investigation confirmed these assumptions. While encouraging clinical results were found in the present case series, along with high satisfaction rates, radiologically no signs of impaired primary and secondary stability as well as loosening were found during follow-up. In none of the cases was further revision surgery needed leading to a survival-rate of 100% at last follow-up.

Currently, various short-stem designs are available, providing distinct differences regarding level of osteotomy, stem length, and insertion technique [16]. The optimys short stem, which was used in the present case series cannot be easily classified, because it can be both metaphyseal anchoring and diaphyseal anchoring, depending on the individual stem alignment according to the patient’s anatomy [20, 21]. Regarding the successful achievement of sufficient primary stability in revision THA, the design properties, given the individualized meta-diaphyseal anchorage, may therefore account for advantages compared to alternative short-stem designs. It allows for a fit-and-fill in the proximal diaphysis, if desired.

In the literature, the use of primary stems for revision THA is only possible under certain conditions: only mild proximal bone defects and the possibility of obtaining perfect primary stability [15, 27]. These conditions were mostly met in our series. Except for one case, bone defects before and after exchange were stages I or II according to Paprosky [34]. Although in our series a femoral revision using a short stem was successfully performed in one case with a Paprosky type IIIa defect, we cannot recommend this conduct routinely. In that context, longer distal fixation may be preferable as is the case for more severe defects [24].

In general, revision surgery using a short stem should not be considered as the standard procedure but more as a salvage procedure, in case other treatment options are either not possible or would lead to disproportionally high risks for the patients. Each decision to use a short-stem design in the present case series was controversial, made after consideration of limited and high-risk options.

Although our series was limited by the small number of cases as well as the short follow-up, making it difficult to draw firm conclusions on the durability of fixation, our main goal was to determine the feasibility and morbidity of this procedure. Revision surgery using short-stem THA is scarce as it should not be the standard procedure and is considered off-label use. Therefore, small series as well may play an important role providing new insights to the orthopedic community; however, long-term follow-up is needed as it is not yet known, if ingrowth occurs unmitigated in a sclerotic bone revision scenario. Since in the present series only one particular short-stem design was used, however, the results cannot be simply transferred to deviant further short-stem designs.

## Conclusion

Based on the present data, confronted with limited options in certain assorted cases, “downsizing” the femoral component may be considered as an alternative. The preservation of most of the metaphyseal femoral bone stock after primary implant extraction is a mandatory requirement. Revision surgery using a short stem should, however, not be considered as a standard procedure and should be reserved for experienced surgeons.

## Abbreviations

EQ-5D-5L: Health status by the EuroQol Group
HHS: Harris Hip Score
HRA: Hip resurfacing arthroplasty
THA: Total hip arthroplasty
VAS: Visual analogue scale
WOMAC: Western Ontario and McMaster Universities Osteoarthritis Index

## Declarations

All procedures performed in studies involving human participants or on human tissue were in accordance with the ethical standards of the institutional and/or national research committee and with the 1975 Helsinki declaration and its later amendments or comparable ethical standards. Prior to study initiation, all patients provided written informed consent for the study and publication of anonymized data. IRB: The present study was approved by the local ethics committee (Landesärztekammer Hessen (Approval No. 2019-1164-evBO)) prior to patient inclusion.
